# Anæmia and Anæmiac Palpitation

**Published:** 1865-12

**Authors:** David Wooster


### ANÆMIA AND ANÆMIAC PALPITATION.—PHYSIOLOGY OF THE BLOOD—ABSTRACTS AND COMMENTS.

By DAVID WOOSTER, M.D., ETC.

Anæmia has been long known to be associated with palpitation of the heart, but precisely how it is the cause of palpitation, is not clearly understood. Properly speaking, there is but one cause of palpitation, and that is lesion, either functional or organic, of the pneumogastric nerve, which is the acknowledged regulator of the heart's rythmical motion. Anæmia, to affect the heart's rythm, must first affect the pneumogastric nerve, and hence the study of palpitations becomes a study of innervation or ganglionic nutrition, and this leads us directly to the investigation of the physiology of the blood itself. Anæmia is by no means a word expressing a definite pathological condition. For example: the blood is composed of red and white globules and of plasma; the plasma consists of fibrine and serum; the serum, of albumen, certain salts and water. By anæmia we surely do not mean a deficiency, or lack even, of all these substances; for, on the contrary, in the disease recognized as anæmia there may be merely diminution of red globules—this would constitute *globulia* only; there may be lack of albumen, which would constitute an *albumenœmia*—and so with all the constituents which go to make up that heterogeneous current of evil and good we call blood. It will be remembered, blood is not merely material of which organs are to be made and repaired: it contains not merely living, but also dead matter. In its purple current are mingled the products of destructive metamorphosis and the elements which are to repair this destruction and waste. The employment of a concrete term to express several discrete facts is not scientific.

When the mass of the blood is considerably or repeatedly diminished, when the globules are relatively or absolutely diminished, when the watery part of the plasma becomes excessive, when the albumen of the serum falls below its physiological limit, there occur a set of symptoms which we call anæmia. In this sense, chemically and histologically, anæmia is total, globular, albuminous or hydremic; defect of albumen rarely exists alone; it is followed by consequences at once peculiar and dangerous. The excess of water in the blood is almost always long subsequent to the diminution of globules; it is a very advanced stage of general anæmia. The first pathological condition in anæmia is defect of globules only.

The causes of anæmia exist not in the blood itself, but are various as the means by which the equilibrium of the principles of the blood is maintained; as various as the histo-chemical elements employed in its genesis: as various as the organs or tissues which preside over the formation, destruction, and regeneration of the plastic or globular elements.—(Lee.)

The blood neither lives nor changes itself. There is no such thing as a blood disease. It is merely a secondary alteration, depending on some lesion of nutrition or assimilation. The disease we call anæmia is an affection in which the whole organism takes an active part, either through the generic influence of the organs upon the composition of the blood, or through the changes which the altered blood in its turn impresses on the various functions.

As we said above, the blood is not an independent thing—it gives and receives despite itself. The organs and tissues, as Claude Bernard has shown, appropriate by elective affinities the principles of the blood. The blood does not contain all the substances found in the secretions or in the parenchyma of organs; neither gastric juice, bile, nor the genital secretion is found in the blood. The blood furnishes merely the primitive materials; the organ elaborates and modifies them according to its peculiar function. The organ is active, the blood passive. The latter receives indiscriminately, and restores in the same manner, the products of nutritive metamorphosis, and generally in the most simple forms. Of all the elements composing the blood, but one presents a true autonomy: this is the globule.—(Virchow.) The physiologist recognizes in the blood only two essential parts: the globules and interglobular liquid, or plasma.

Plasma deprived of globules and fibrine constitutes serum—

contains, 1st. Albuminous substances (which can only become cause of disease by diminishing so as to alter the physical fitness of the blood.—(C. BERNARD.) 2d. Products of protein bodies. 3d. Flats; and finally, inorganic matter and salts and water.

The red and white globules are the only elements of the blood endowed with vitality. The globules have been compared to living cells: they live, perform their function, and die in the midst of the plasma. However intimate the relation between the plasma and the globules, they possess properties altogether distinct. The plasmic elements may pass into the secretions and tissues; in fact, the entire plasma, except the fibrine, may, under special conditions, become infiltrated into the surrounding tissues; but a globule never passes through the walls of the capilliaries in its physiological condition. Bischof says the transfusion of plasma without globules is exceedingly dangerous, and, in any considerable quantity, fatal, whereas the transfusion of globules without fibrine (C. Bernard says) will often restore life to the really dead. The globules are clearly indispensable to the functions of the organs. They are the vital elements of the blood just as cells are of the tissues.

Schwan says the globule has an envelop, a viscous and colored contained substance, and a nucleus. The nucleus is now given up and acknowledged to be an illusion. Schultze denies the envelop, and says the appearance is the result of greater density of the globule at its periphery. The nucleus is only a depression in the bi-concave disc, indicated by a semi-circular shadow and clear space, this dark depression has given rise to the belief in the existence of a central nucleus. Bruelke says the globules have no envelop: that it does not follow because they lose their discoid form in water and become spheroidal, that therefore they have a special membrane. A nucleus is not necessary to the existence of cell; the function of the nucleus is important during the division of the cells and endogenous formations, but its intervention may be omitted and the division proceed directly from the *protoplasma*, which penetrates both the nuclear mass and the fragment.

Schultze denominates *protoplasma*, that substance in which the nuclei repose, including the nuclei. Thus the mass in which repose the nuclei of striated muscular fibres, may be considered the protoplasma of cells, although this matter may be merely finely dotted, and not separated by any membrane from the morphosis of the protoplasma of the contractile muscular substance. Connective tissue is developed in the same manner at the expense of the protoplasma. The intercellular substance of this tissue does nor result from division or budding of another cell; but it is the product of a continual metamorphosis of the protoplasma of those cells which are in process of growth. The moment the protoplasma loses its vitality it becomes an inert substance soluble in water, and this condition is recognizable by the absence or decrease of nuclei. A blood globule which has no apparent nucleus (depression) is a dead globule, no longer fit for purposes of nutrition. This is the theory of Schultze, but Bruelke says the nucleus itself is *not* indispensable to the constitution of the cell and its reproduction, as remarked above. He says the protoplasma itself possesses the property of contractility. This contractility is visible under three forms in the organism. 1st. Contractility in the striped muscles, that is, in their protoplasm. There is another species of contractile substance found only in inferior organisms—the infusoriæ, known as *amibes*, seem to be composed entirely of this contractile substance, which is called *sarcode* (flesh like), and which coagulates at 80° F. Kuhne found this substance in the cornæ of frogs, and in pigmentary cells. A third species of contractile movements is found in the vibratile ciliæ, which line the respiratory organs and the inter nal genital organs of woman. Then there is a molecular movement in the protoplasm of a multitude of cells entirely distinct from muscular fibro-cells. Virchow observed this motion in white globules, Recklinghausen in purulent and mucous globules which are able, by progessive movements, to reach the surface of the membranes.

This is quite distinct from molecular displacements, and is entirely suspended on the death of the cell. If an inductive current be made to pass through these cells the molecular movement ceases, the cells suddenly collapse, exhibiting only nuclei, contraction seems then to depend upon vital manifestation.

The real texture of the red globule may be considered as settled beyond cavil. It is not a vesicle composed of a special membrane. It is composed of a substance which is extensible, very elastic, susceptible of assuming almost any shape under external forces, and of resuming its original discoid form when the force is removed. This is proved by the experiment of Rollet with a concentrated solution of gelatine; in which globules had been artificially brought together; gelatine so charged is partially dried and submitted to pressure under the microscope, when the globules are seen to assume all forms, and even to rupture and disappear on being distended with the solution; but in no case is a collapsed membrane seen, nor any contents ever seen to escape from a vesicle.

Congelation of the globules repeats these phenomena; the depressed portion or nucleus of the globule remains visible longer than the surrounding portion. Static electricity acts like congelation: the globules are for the most part destroyed, and in the blood of some animals hematine crystals are deposited at the same time. Ether produces analogous results: the blood grows clear, the globules smaller and they disappear without rupture.

Finally, it has been demonstrated by Bruelke, Rollet, and more recently by Klebs and Alex. Schmidt, that red globules are contractile bodies, whose contractility may last some hours after death, and that this contractility depends solely upon the *protoplasma*.—(LEE.) All this exactness of research on the physiology of the blood has much to do with the exact diagnosis of disease, and with the simplification of nosology. It will be manifest that treatment must in the main be nutritive—histochemically speaking—and that waste is quite as essential as reparation; or in other words, morphosis and metamorphosis must be equally active.

I am of the opinion that if we study farther we shall find globular lesions to occupy a much more extended range in the production of disease, than has hitherto been attributed to them. Anæmia is a generic term, including many morbid changes, as well as being the exponeat of a well-recognized state of the organism. But it must be borne in mind that the lesion of the globule is not primary, and indeed can hardly be conceived to originate in the globule itself. The altered globules will die—let them, they cannot be restored; but treatment can induce the organism to replace them with normal globules, and to increase the number of the latter, and this will in turn restore the altered functions, and the latter will in turn assist the re-formation of the defective blood. Nothing is more detrimental to the integrity of the blood than continued pain, or mental or moral disturbance; a quite mind in a body free from pain is the best restorative of the blood; and producing and maintaining this condition is the most speedy way to cure *anæmia* and anæmiac palpitations.—*Pacific Med. & Surg. Jour.*

